# Convergent genomics of longevity in rockfishes highlights the genetics of human life span variation

**DOI:** 10.1126/sciadv.add2743

**Published:** 2023-01-11

**Authors:** Stephen Treaster, Joris Deelen, Jacob M. Daane, Joanne Murabito, David Karasik, Matthew P. Harris

**Affiliations:** ^1^ Department of Orthopaedic Surgery, Boston Children’s Hospital, Boston, MA, USA.; ^2^ Department of Genetics, Harvard Medical School, Boston, MA, USA.; ^3^ Max Planck Institute for Biology of Ageing, Joseph-Stelzmann-Str. 9b, D-50931 Köln, Germany.; ^4^ Molecular Epidemiology, Department of Biomedical Data Sciences, Leiden University Medical Center, Leiden, Netherlands.; ^5^ Cologne Excellence Cluster on Cellular Stress Responses in Aging-Associated Diseases (CECAD), University of Cologne, Cologne, Germany.; ^6^ Department of Biology and Biochemistry, University of Houston, Houston TX, USA.; ^7^ Section of General Internal Medicine, Department of Medicine, Boston University School of Medicine, Boston, MA, USA.; ^8^ Framingham Heart Study, Framingham, MA, USA.; ^9^ Azrieli Faculty of Medicine, Bar-Ilan University, Safed, Israel.; ^10^ Marcus Institute for Aging Research, Hebrew Senior Life, Boston, MA, USA.

## Abstract

Longevity is a defining, heritable trait that varies dramatically between species. To resolve the genetic regulation of this trait, we have mined genomic variation in rockfishes, which range in longevity from 11 to over 205 years. Multiple shifts in rockfish longevity have occurred independently and in a short evolutionary time frame, thus empowering convergence analyses. Our analyses reveal a common network of genes under convergent evolution, encompassing established aging regulators such as insulin signaling, yet also identify flavonoid (aryl-hydrocarbon) metabolism as a pathway modulating longevity. The selective pressures on these pathways indicate the ancestral state of rockfishes was long lived and that the changes in short-lived lineages are adaptive. These pathways were also used to explore genome-wide association studies of human longevity, identifying the aryl-hydrocarbon metabolism pathway to be significantly associated with human survival to the 99th percentile. This evolutionary intersection defines and cross-validates a previously unappreciated genetic architecture that associates with the evolution of longevity across vertebrates.

## Teaser

The genomics of longevity in rockfish and humans reveals shared signatures in the same gene set, aryl-hydrocarbon metabolism.

## INTRODUCTION

Aging pathologies may be delayed, ameliorated, or prevented in aggregate by targeting the molecular foundations of the declines in homeostasis and function that arise over time. The knowledge of foundational targets suitable for such intervention remains limited, yet evolution has already leveraged such means, as is evident in the vast diversity of longevities in nature. Various species display aging-associated functional declines at wildly different rates and timings, including those that survive well beyond a human life span. As these traits are heritable and defining for many species, the underlying genetic mechanisms can be tracked through comparative genomic approaches.

Research into the genetic regulation of longevity has primarily focused on model organisms with life spans on the order of weeks, months, or a few years, enabling broad screens and timely study of experimental interventions. However, short-lived organisms represent a fundamentally different evolutionary strategy, and the idiosyncrasies influencing their aging may not apply to longer-lived models, including humans (*1*, *2*). The effects of the best dietary, pharmaceutical, and genetic manipulations in model organisms are far outclassed by the life span variation between species. Despite great strides in unraveling mechanisms of aging in experimentally accessible rodent models, they are still limited to approximately 20 to 50% life extension when targeting canonical aging pathways (*3*). A more extensive understanding of the regulation of longevity may require analyses of long-lived models.

**Fig. 1.**
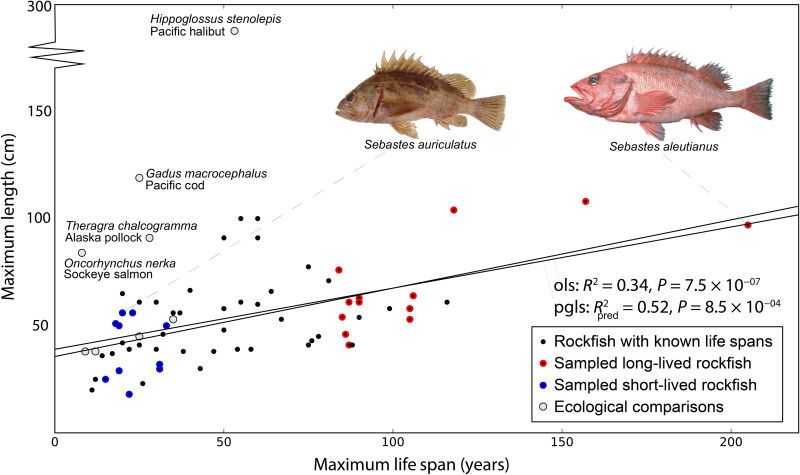
Rockfish exhibit a 20-fold range in longevities. Available maximum longevities and lengths and for 65 rockfish species (black dots) and ecological comparators (gray dots). While there is a significant relationship between size and longevity, it only explains about half of the 20-fold range in longevities, with substantial overlap in size across the spectrum. This capability is exceptional, as *S. aleutianus* can survive for over 200 years, while the much larger Pacific cod and halibut survive for only 25 and 55 years, respectively, despite having overlapping ecologies. The phylogenetic generalized least squares (pgls) regression includes only the short- and long-lived rockfish sampled and sequenced here, corrected with the phylogeny derived below. For illustrative purposes, the ordinary least squares (ols) regression includes all rockfish plotted with known lifespans.

**Fig. 2.**
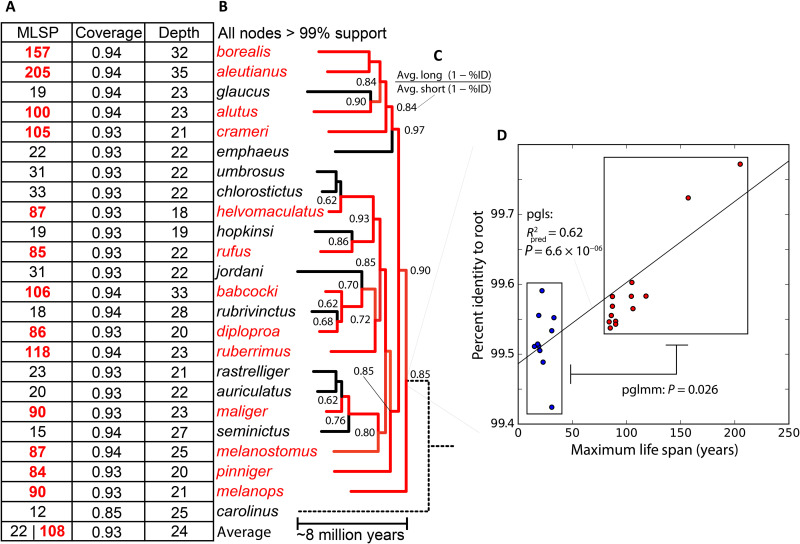
Targeted genomic sequencing of rockfishes with multiple independent reductions of longevity. (**A**) Targeted capture and sequencing from short- (black) and long-lived (red) rockfish, with average maximum life spans (MLSP) of 22 and 108, respectively. Approximately 93% of the targeted exons and CNEs were reconstructed at an average depth of 24×, covering an average of 43,120,434 bases. Less than 100% coverage is expected, as many elements will be unique to reference genomes used and/or lost in rockfish evolution. (**B**) Rockfish phylogeny generated from a set of high coverage elements with support values greater than 99% at each node. (**C**) Node values here are the ratio of the average long-lived to average short-lived percent identity to the reconstructed ancestral sequence at that node. This value is always below 1.0, indicating that the substitution rate is inversely related to longevity across the phylogeny. (**D**) At the rockfish root, where every species can be compared, there is a significant linear relationship (pgls, phylogenetic generalized least squares) and group difference (pglmm, phylogenetic generalized linear mixed model) between longevity and percent identity to the ancestral state.

There are many examples of long-lived animals that have evolved to endure the rigors of time, such as the 507-year-old bivalve *Arctica islandica* (*4*), nearly 400 years for the Greenland shark (*5*), and over 200 years for bowhead whales (*6*). The Rougheye Rockfish, *Sebastes aleutianus*, is one such vertebrate species, with a maximum life span of over 205 years (*7*) as determined from growth ring annuli in otoliths (*8*). Regardless of the aging mechanism—oxidative damage, proteostasis collapse, DNA damage, telomere/genomic maintenance, epigenetic drift, etc.—*S. aleutianus* resists the deleterious effects of age for over two centuries, enduring the variety of internal and external stressors assured with time (*9*, *10*). *S. aleutianus* is not the only rockfish lineage with this exceptional capability. The clade encompasses at least 107 extant species, ranging in maximum longevity from 11 to 205 years (Fig. 1) (*7*). Fortunately, multiple, independent lineages of rockfishes exhibit impressive life spans (Fig. 2B), imparting power into comparative approaches (*11*, *12*). These approaches are further augmented by the relative youth of the *Sebastes* clade, emerging about eight million years ago, as calibrated by molecular clocks and supported with well-characterized paleo-geographic landmarks (*13*). This recent divergence helps to minimize genetic changes between species, such that identified differences between the short- and long-lived lineages are more likely to be facilitating that phenotypic change.

Critically, variation in rockfish longevity is only weakly correlated with size (Fig. 1) or with ecological determinates such as temperature and depth (*14*). The independence from common longevity correlates minimizes the contamination of putative longevity mechanisms with those facilitating the other traits (*15*). This independence is especially evident in ecological comparisons; comparator fish lineages rarely make it past two decades, even those that reach substantially larger sizes and live in overlapping environments (Fig. 1). The 2.53-m, 363-kg Pacific halibut is one of the largest fishes living in these waters, yet only reaches a quarter of the life span of *S. aleutianus*. Rockfish longevity is truly exceptional, and their diversity, availability, and recent divergence combine to provide a unique opportunity for comparative genomics to elucidate the genetic mechanisms of phenotypic change (*13*), particularly the regulation of longevity.

The conservation of genetic mechanisms between vertebrates (*16*, *17*), particularly those regulating aging (*18*, *19*), suggests that the mechanisms underlying exceptional longevity may be consistent across species. This concept empowers meta-analyses, where broad evolutionary comparisons of exceptional longevity, within and between species, can refine our knowledge of fundamental mechanisms (*20*). Here, we leverage the rich diversity of rockfish longevities along with the extensive genome-wide association data available from long-lived humans (*21*). The intersection of these approaches reveals conserved targets that are linked to extended vertebrate life span.

## RESULTS

### Targeted capture, sequencing, assembly, and rockfish phylogeny

Modern methods for convergence analyses rely on high-quality, annotated genomes with dense taxonomic sampling. This requirement often precludes analysis of novel, poorly studied groups (*22*). Such investigations would necessarily lack genomic resources of sufficient quality and taxonomic breadth to resolve trait-associated variation from other shared or species-specific variation. This is particularly problematic, yet prevalent, in the investigation of exceptional longevity; most notable occurrences are singular events, either in one isolated lineage or shared by the entire group (*23*, *24*). The consequence is a shortage of meaningful comparisons to subtract variation unrelated to the trait of interest. The *Sebastes* genus, with their multiple independent losses of exceptional longevity, provides such comparisons within its evolutionary history.

To capitalize on *Sebastes*’ natural experiment in modulating longevity, we expanded upon our recently developed phylogenomic mapping strategy (*25*), permitting broad sampling and sequencing of novel lineages with minimal established genomic resources. Here, we specifically targeted 23 rockfish species, representing the shortest-lived (average 22 years) and longest-lived extremes (average 108 years) and encompassing eight independent instances of trait change. We purposefully focused our sampling at the extremes of the rockfish longevity spectrum and binned short- and long-lived as a binary trait. There is no apparent bias in the trait distribution of the lineages sampled. Should they exist, our downstream comparative analyses specifically correct for these issues. The lineages were chosen over those whose maximum life span or phylogenetic relatedness would not particularly inform comparative analyses. This sampling minimizes the risk of misphenotyped lineages and ensures that corrections for body size or ecological correlates will not change the binary trait binning that is required for downstream analysis. The *R*^2^_pred_ (*26*) suggests that only half of the variation in life span in these species can be predicted by body size alone (Fig. 1).

We targeted sequencing using a genome-wide, pan-perciforme oligoarray containing 285,872 coding elements, 118,406 conserved noncoding elements, 2508 ultraconserved noncoding elements, and 298 microRNAs (miRNAs). These targets were derived from broad synthesis of available genomic data from close relatives (fig. S1). Using the well-annotated Stickleback, Medaka, and Green Spotted Puffer genomes as the core reference, we recovered and reconstructed an average of 93% of conserved elements (coding and noncoding) at an average depth of 24×, spanning an average of 43,120,434 bases. It should be noted that less than 100% coverage is expected, as many elements will be unique to the reference genomes and/or lost in rockfish evolution. Genes unique to rockfish will also be missed in this approach. However, the assembled elements will represent conserved functionality that is more likely to inform and apply to longevity mechanisms outside the genus. As pooled populations were used for each species, both fixed and variable alleles for each were identified (Fig. 2A and table S2). As an outgroup, the northern sea robin *Prionotus carolinus* was included to root our analyses.

To generate a species tree for comparative analyses, a set of universal, high-coverage exons were concatenated, spanning 6,530,274 bases, and used to generate a phylogeny with IQTree using model finder (*27*). All nodes have greater than 99% bootstrap support (*28*), resolving rockfish evolutionary relationships and providing a valuable foundation for downstream analyses (Fig. 2B). The major groupings in this phylogeny are consistent with previous analyses (*13*).

### Longer-lived Rockfish lineages have fewer changes from the ancestral state

Given the phylogeny of the rockfish clade and the prevalence of such otherwise rare longevities within the genus and the greater *Sebastidae* family, we hypothesize that the evolution of longevity-extending mechanisms likely arose early. These mechanisms would then be shared among long-lived rockfish, yet have been independently lost in the many short-lived lineages. The pattern of branch lengths on the phylogeny hint that long-lived species may be genetically more similar to the ancestral state as compared to the short-lived, supporting this hypothesis. To quantify this pattern, ancestral sequences were reconstructed with IQTree, and the percent identity of each terminal branch was calculated as compared to each node ancestral to that branch. At every node, the average percent difference of the long-lived descendants was lower than the short-lived, represented as a consistent ratio below 1.0 (Fig. 2B). At the root, where all species are included, there is a significant linear relationship between longevity and percent identity [phylogenetic generalized least squares (pgls), *P* = 6.6 × 10^−6^]. This relationship is also significant when bundling the short- and long-lived species as a binary trait [phylogenetic generalized linear mixed models (pglmm), *P* = 0.026] (Fig. 2C). As the long-lived lineages are more similar to the ancestral state than the short-lived, these data support the evolution of extended longevity occurring early in the stem rockfish and subsequent loss in independent lineages. However, inferences based solely on substitution rates can be confounded by interactions with variability in germline mutations accumulating with individual age (*29*), changes in time to reproductive maturity, number of generations, and population size (*30*). These metrics are only sparsely available for these populations, and thus these covariates cannot be currently addressed except to rely on indirect inferences.

### Specific genes and gene sets are convergently evolving with longevity shifts in rockfishes

**Fig. 3.**
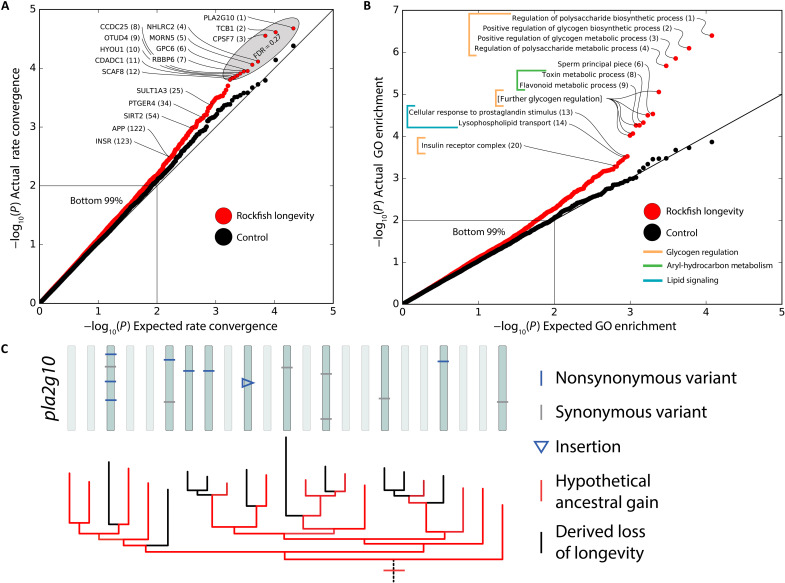
Convergent rate analysis reveals specific genes and gene sets under convergent evolution with rockfish longevity. TRACCER identifies convergent shifts in the evolutionary rate of specific genes (**A**) and gene sets (**B**) associating with longevity in rockfish. Both are enriched at low *P* values, indicated as the displacement between the red experimental line and black control. The top 12 genes in total have an FDR of 0.27. GO enrichment of the gene results reveals significant gene sets evolving with longevity, including numerous overlapping terms related to glycogen biosynthesis (*q* = 0.005) and aryl-hydrocarbon (flavonoid) metabolism terms (*q* = 0.073). Because of the overlap in gene sets using propagated (indirect) annotations, these *q* values should be interpreted as conservative. (**C**) Analysis of variation in *pla2g10* highlights the independent accumulation of mutations in short-lived lineages (black), while long-lived lineages (red) maintain the ancestral state without change in every lineage but one.

The percent identity pattern indicates that genome-wide evolutionary rate shifts have been altered alongside longevity. We next asked whether these shifts were enriched at specific, functional loci, identifying them as putative longevity regulators. The captured and sequenced coding regions were concatenated into genes, and gene trees were generated with IQTree. These gene trees were analyzed with TRACCER to quantify convergent rate shifts associated with longevity (*31*). Importantly, this comparative method takes phylogenetic relationships into account without ancestral state assumptions. As compared to a control set of species balanced for similar phylogenetic relatedness, in which half the traits were inverted, the analysis showed enrichment for genes with significant rate shifts associated with longevity (Fig. 3A). The control selection has a slight enrichment as well, likely due to the difficulties in selecting an unbiased control group when all the lineages were explicitly sampled for their extreme trait status. Critically, when these signals are expanded upon with Gene Ontology (GO) enrichment, the control signal is muted. Using propagated GO annotations (indirect included), we find a dramatic enrichment at low *P* values for longevity, while the control group matches the distribution expected by chance (Fig. 3B). Including the indirect annotations entails substantial overlap in many gene sets, such that they are not independent tests and multiple hypothesis corrections should be interpreted as extremely conservative. To help minimize redundant terms and increase specificity for downstream analyses, we also ran the enrichment using only direct annotations, recapitulating the same functional results (table S5). These results indicate not only that specific genes are being maintained with longevity, but also that these patterns underly specific functions. We similarly analyzed conserved noncoding elements we recovered in our targeted capture; however, the rate-based approach used here was underpowered to parse rate differences given their small size and high levels of conservation.

The top genes identified in this analysis include *pla2g10*, *tcb1*, *cpsf7*, *nhlrc2*, *morn5*, *gpc6*, *rbbp6*, *ccdc25*, *otud4*, *hyou1*, *cdadc1*, and *scaf8*. While individually, they are only marginally significant, the collection is enriched and, in total, have a false discovery rate (FDR; *q* value) of only 0.27, representing the major FDR inflection point (Fig. 3A and table S3). Interestingly, these genes are not driving the signal in the identified gene sets, which include glycogen biosynthesis, aryl-hydrocarbon metabolism, lipid signaling, and sperm descriptors (Fig. 3B and table S4). These gene classes were recapitulated using direct annotations, specifically identifying “positive regulation of glycogen biosynthesis” (*q* = 0.019) and “flavonoid metabolism” (*q* = 0.009) (table S5). Both gene sets are undergoing evolutionary constraint in long-lived lineages or, equivalently, accelerated sequence evolution in the short-lived lineages.

To determine how these patterns map to selective pressures, we ran HyPhy aBSREL (*32*) and RELAX (*33*) on concatenated gene sets, comparing the short-lived lineages to the background rates on the rest of the tree. For the flavonoid pathway, aBSREL detected diversifying positive selection in 8 of 10 short-lived lineages: *Sebastes jordani*, *glaucus*, *seminictus*, *umbrosus*, *rasterlliger*, *rubrivinctus*, *auriculatus*, and *chlorostictus* (*P* = 0.00062, 0.00868, 0.00046, 0.00002, 0.00008, 0.00111, 0.01342, and 0.03016 respectively). RELAX detected intensified selection across the short-lived lineages (*k* = 1.50 and *P* < 0.0001). We find a similar pattern in the glycogen pathway, with RELAX again identifying intensified selection across the short-lived lineages (*k* = 1.23 and *P* = 0.0022), while aBSREL detected diversifying positive selection in three short-lived lineages: *Sebastes emphaeus*, *rastrelliger*, and *rubrivinctus* (*P* = 0.0363, 0.00033, and 0.01184 respectively). In conjunction with the relative evolutionary rates TRACCER detected in these gene sets, these results indicate that the short-lived lineages are convergently adapting the flavonoid metabolism and glycogen biosynthesis pathways to facilitate their reduction in life span.

As a proof of concept of our approach and longevity dataset, the signals we find share commonalities with established aging and longevity studies. The glycogen biosynthesis term has substantial overlap with insulin signaling pathways, and indeed the most significant gene in the set is insulin receptor (*insr*) (*P* = 0.003). Insulin signaling has been implicated in the aging process and longevity in a wide variety of models (*34*, *35*). *Insr* was one of the genes identified as undergoing relaxed selection in the evolution of shortened longevity in killifish radiations (*36*), i.e., the same gene is undergoing similar evolutionary pressures in independent examples of shortened longevity.

Beyond insulin signaling, we identified several genes underlying the evolution of rockfish longevity that have been implicated in aging in diverse models. These include the 54th ranked gene, *sirt2* (*P* = 0.001) (*37*), and the 122nd ranked *app* (*P* = 0.003). The latter is the source of amyloid fibrils in Alzheimer’s disease, yet it is also an ancient and conserved gene involved in diverse signaling pathways (*38*), including reproduction and hormonal signaling (*39*). Notably, the sixth ranked gene, *gpc6* (*P* = 1.12 × 10^−4^), is associated with increased life span in human populations (*40*, *41*). The highest-ranked gene for convergent evolution with rockfish longevity is *pla2g10*, with the short-lived rockfish lineages accumulating unique variants, while the long-lived lineages retain the ancestral rockfish sequence (Fig. 3C). *pla2g10* encodes a secreted phospholipase (sPLA), which has not been previously associated with aging or longevity. Intriguingly, sPLA2s bind to PLA2R1, which has been described as the “master regulator of cellular senescence,” acting through reactive oxygen species, DNA damage, and p53 (*42*–*45*). Thus, clear candidates were identified convergently evolving in rockfish that highlight both known mechanisms (glycogen/insulin signaling) and novel mechanisms (flavonoid/aryl-hydrocarbon metabolism) underlying the evolution of longevity.

### Rockfish longevity results provide new pathways to highlight human longevity GWAS

Previous efforts to associate loci with the regulation of longevity in humans have been hindered by the complexity of the trait and the statistical burden of multiple hypothesis testing across the genome. Given the conservation of core mechanisms among vertebrates, we used the resulting genes and gene sets from the rockfish analysis as enriched windows to explore the largest genome-wide association study (GWAS) of human longevity to date (*21*).

**Fig. 4.**
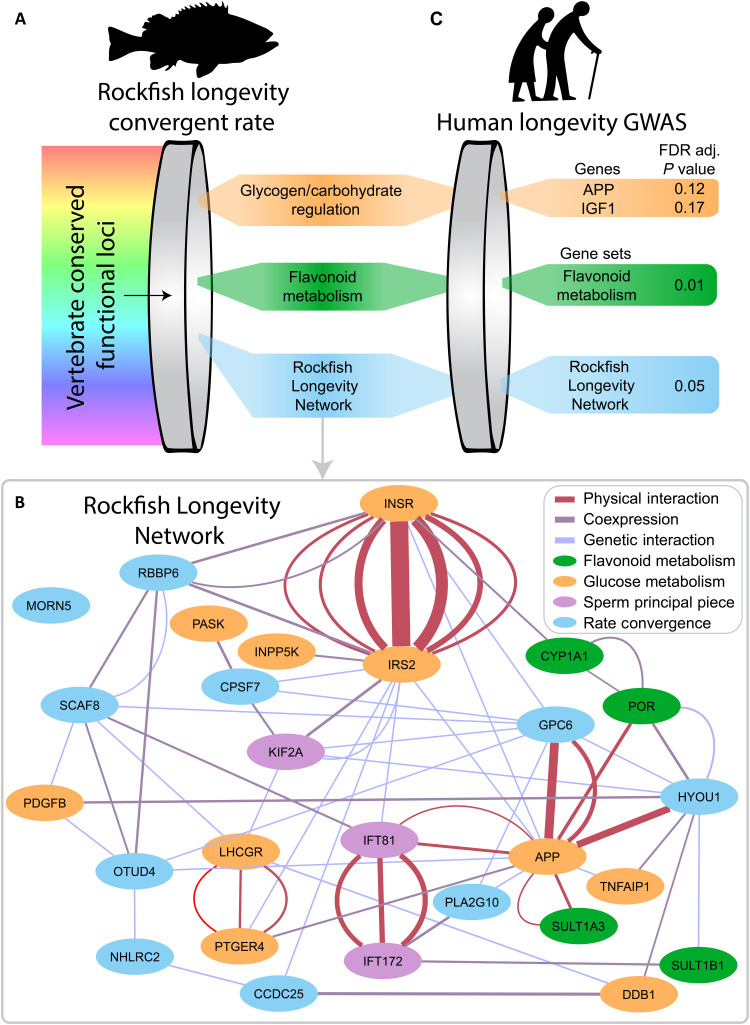
Rockfish longevity genes are enriched for cross-function interactions and contain SNPs associating with human longevity. (**A**) Convergent rate analysis of rockfish coding regions reveals enrichment of genes involved in flavonoid metabolism and glycogen biosynthesis. (**B**) Network analysis of the top rockfish convergence genes (*q* < 0.27) along with the top genes (*P* < 0.1) in the top indirect GO terms (*q* < 0.1) demonstrates extensive interactions, including across terms. For instance, APP physically interacts with IFT81, POR, and SULT1B1, representing the other enriched indirect GO terms, along with GPC6 and HYOU1, which are not characterized in these terms. Individual lines are individual experiments confirming that interaction. (**C**) When human GWAS of longevity is focused upon the top gene sets from the rockfish longevity analysis, the multiple hypothesis testing is dramatically reduced. The flavonoid metabolism gene set is associated with human survival to the 99th percentile (*q* = 0.01), while the custom "Rockfish Longevity Network" is associated with survival to the 90th percentile (*q* = 0.05).

Because mapping the functionality of orthologs across vast evolutionary time can be problematic, we used the rockfish-enriched GO sets to directly translate functionality between species. The significant, directly annotated terms were used to minimize redundancy, including flavonoid metabolism and glycogen biosynthesis (Fig. 4A). In addition, we made a custom gene set defined using the top TRACCER genes with a combined FDR of 0.27, along with only the most significant genes (*P* < 0.1) within the enriched indirect GO terms (*q* < 0.1) (fig. S2 and table S5). Network analysis on this “Rockfish Longevity Network” with GeneMANIA (*46*) demonstrates substantial interactions across functional groups (Fig. 4B). For instance, amyloid precursor protein (APP), one of the genes driving the significance of the carbohydrate metabolism gene set, physically interacts with members of each of the functional groups. These interactions suggest that the convergence rate analysis here has resolved an extended genetic architecture related to longevity.

These gene sets were intersected with results from the recent worldwide GWAS for human longevity by Deelen *et al.* (*21*). While the glycogen term was not significant, single-nucleotide polymorphisms (SNPs) within the flavonoid metabolism gene set were significantly associated with human survival to the 99th percentile (*q* = 0.01). Furthermore, we identified the custom "Rockfish Longevity Network" with survival to the 90th percentile (*q* = 0.051), while *APP* (*q* = 0.12), *IGF1* (*q* = 0.17), *LHCGR* (*q* = 0.31), *C1QTNF2* (*q* = 0.32), *SULT1B1* (*q* = 0.36), *MORN5* (*q* = 0.41), and *PTGER4* (*q* = 0.50) within these sets were modestly associated with human survival to the 90th or 99th percentile, although the strongest signals were at the set level (Fig. 4C and tables S6 and S7). These shared signals in both rockfish and human cases of exceptional longevity indicate a conservation of longevity regulators across vertebrates.

## DISCUSSION

There is conserved genetic architecture shared by all vertebrates (*16*, *17*), including that which outlines longevity (*18*, *19*). Nutrient signaling and growth pathways have been implicated as life span factors across Metazoa, but the specific changes that set the arc of a dwarf goby’s life to only 60 days (*47*), a mouse’s to 4 years, and a human’s to roughly 80, have remained undefined. These life-history traits are shaped in evolution through changes in genetic controls, but disentangling these key variations from other species-specific changes is infeasible using isolated species genomes. However, with independent evolutionary occurrences and analyses capable of bridging diverse genetic contexts, shared signatures within genomes can be identified across species (*20*). Here, we leverage the exceptional longevity and diversity of rockfishes to identify gene sets underlying the evolution of longevity. We then cross-validate those gene sets with variation associating with longevity in human populations, providing an independent test of their core function in modulating longevity.

Fish longevities past 100 years are exceedingly rare, yet are common in the rockfish clade and the greater *Sebastidae* family, suggesting that the ancestral state of this group may also be long lived. Our analyses revealed genome-wide evolutionary rate shifts alongside changes in longevity; long-lived lineages have fewer changes overall and are more similar to reconstructed ancestral sequences than the short-lived lineages. This trend in percent identity entails an increased substitution rate in the short-lived lineages, a pattern that has been demonstrated in rockfish previously when looking at genome-wide nucleotide diversity across the clade (*12*). Other radiations with variable longevities, such as killifish, show the same pattern as well (*36*). This is likely due to reduced investments in germline maintenance as reproductive maturity accelerates (*48*), although the influence of generation counts and population sizes (*30*) may complicate the relationship. The ecological pressures that influence longevity are certainly entangled with those that determine maturation timing, although the specific costs related to these life-history traits in these ecologies are unknown.

The ancestral state of exceptional longevity in rockfishes is reinforced by our convergence analysis; specific loci and functional gene sets are convergently undergoing relative rate shifts, maintaining the ancestral state in long-lived lineages or, equivalently, accelerating sequence evolution in the short-lived lineages. These signals were initially identified by TRACCER, which has the key feature of remaining agnostic to ancestral state (*31*). Informed by those results, we targeted our subsequent selection analyses to short-lived terminal branches, revealing significant diversifying selection in these pathways alongside the shortening of longevity. Recent analyses of rockfish genomes (*12*) and transcriptomes (*11*) have assumed or calculated that exceptional longevity is derived. These state reconstruction calculations, based solely on extant characteristics, require an assumption of evolutionary neutrality across the tree, which is inappropriate in a convergent context (*49*). Misattributing ancestral states will reduce downstream sensitivity when used to guide analyses. This includes branch-site positive selection analyses, which may then be mistargeted, or relative rate approaches that hinge upon ancestral state assumptions (*31*, *50*). Those analyses identified DNA-repair genes (*12*) and immune-related genes (*11*) under positive selection on long-lived branches. However, in the context of a long-lived ancestor, these results are better explained by independent refinements in the extended longevity trait, not the inception of that trait per se. Indeed, most of the identified positively selected genes were unique to individual lineages.

Misattributing branches can confound comparisons and shift sensitivity to detect how and where evolution is acting. We further avoid trait-misattribution issues by focusing our analysis to the extremes of longevity within rockfishes. We avoid the midrange longevities, which risk being mis-phenotyped due to insufficient sampling or may be driven more by size or ecological correlates—the latter of which are difficult or impossible to accurately assess or infer in these environments. We instead obviate these issues by focusing on, and binarizing, the extremes. Then, we cross-validate our longevity results with a well-characterized, independent model, humans.

Our rate convergence (TRACCER) and selection analyses (aBSREL and RELAX) resolved two significant gene sets that the short-lived lineages are adapting to facilitate their more rapid life-history traits: glycogen biosynthesis and flavonoid metabolism. Glycogen biosynthesis incorporates the iconic insulin-like signaling (ILS) pathway that can be modulated with caloric restriction. This is a conserved route for life span modulation across models and recapitulates a similar finding in rockfish by Kolora *et al.* (*12*) after correcting for body size and depth.

Originally discovered in forward genetic screens in nematode worms (*51*, *52*), the role of glucose and energy metabolism in aging continues to be refined (*53*) and retains relevance across vertebrates, including mammals (*54*), and humans (*55*–*61*). Recent analysis of rare coding variants in centenarians converge on ILS pathways (*62*). The highest-ranked gene in the glycogen biosynthesis gene set, and driving its significant enrichment in our analysis of long-lived rockfishes, is *insulin receptor* itself. This is an auspicious proof-of-concept result of our rockfish comparative analysis and lends credence to the novel “flavonoid metabolism” gene set that has hitherto been unappreciated in the regulation and evolution of longevity.

Flavonoids are a diverse and abundant chemical group that share a heterocyclic and two phenyl rings. They are plant metabolites, although they have a plethora of cellular consequences in animals, including anti-oxidative, anti-inflammatory, antimutagenic, and anticarcinogenic properties, and can modulate key cellular signaling and enzymatic pathways (*63*). With such a diversity of potencies, it is unsurprising that flavonoids can influence aging and longevity, and many have been tested to that effect (*64*–*66*). However, the “flavonoid” nomenclature masks the broader role of this gene set in detoxification pathways, processing xenobiotic chemicals, and, critically, modulating endogenous signals such as hormones. The aryl-hydrocarbons in flavonoids and related compounds are detected and induce cellular responses to neutralize and remove these active metabolites, with a variety of downstream signaling consequences with both pro- and anti-aging effects (*67*, *68*). The aryl-hydrocarbon receptor (AHR) is the highly conserved master transcription factor for these responses, triggering xenobiotic responsive genes such as *CYP1A* and sulfur transferases (*69*, *70*)—key members of the flavonoid metabolism gene set. Although the precise ligand interactions have proven complex and elusive (*71*), various flavonoids have demonstrated influence on these pathways, which is how the flavonoid gene set derives its name. The downstream effects of these responsive elements include not only detoxification, but also regulation of hormones and neurotransmitters (*72*). This xenobiotic metabolism has been linked to longevity (*73*). The pleiotropic integration of aryl-hydrocarbon metabolism and hormonal signaling will have marked consequences for growth, energy metabolism, developmental timing, and, ultimately, aging (*74*–*76*).

PLA2G10, the highest-ranked gene in our convergent rate analysis, has the greatest ability among sPLAs to hydrolyze phosphatidylcholine, which promotes inflammation by driving arachidonic acid (AA) production (*77*) and elevated eicosanoid levels (*78*). Linking back to aryl-hydrocarbon metabolism, AA can also stimulate AHR (*79*). AA metabolites such as lipoic acid and lipoxins are tied to inflammation and can ameliorate the aging process (*80*, *81*). Both regulation of polyunsaturated fatty acid synthesis and lipoic acid are down-regulated in human elderly, and the resulting imbalance in AA metabolism removes the brakes from inflammatory reactions (*82*). These fatty acid pathways, and their interaction with aryl-hydrocarbon metabolism, define a potential common axis by which longevity may be modulated. The potential of sPLAs as therapeutic targets in cancer, metabolism, and inflammation have already been noted (*83*, *84*). Intriguingly, sPLA2s are entangled with flavonoid metabolism as well, including direct interactions (*85*). sPLA2 decreases in aged skin but can be rescued with hesperidin, a flavonone (*86*). Red ginseng, rich in the flavonoid catechin (*87*), extends life span in flies and up-regulates sPLA2 (*88*).

A utility of evolutionary refined gene sets, as detailed here, is to inform analyses in other models under the principle of conservation. To validate the role of these targets in longevity across vertebrates, we used them to explore the largest GWAS for longevity in humans (*21*). Human GWAS are empowered by massive sampling and accurate characterization, but its ability to trace complex, multifactorial traits has been disappointing (*89*). Signals for longevity routinely fall short of significance due to multiple hypothesis testing across the whole genome and the complexity of the trait. However, by focusing on the genes and gene sets implicated in exceptional rockfish longevity, we were able to do a hypothesis-based exploration of the human GWAS data. Notably, the flavonoid metabolism gene set is again revealed as significant (*q* = 0.01) for association with human longevity, i.e., survival to the 99th percentile. We also identify the custom Rockfish Longevity Network as associating with human survival to the 90th percentile (*q* = 0.05). Within these sets, we identify *APP*, *IGF1*, *LHCGR*, *C1QTNF2*, *SULT1B1*, and *MORN5*, although the strongest signal is at the set level, suggesting the longevity architecture is modulated as a group. These findings cross-validate the rockfish sets through conservation, and considering these targets were all relatively constrained in long-lived rockfish lineages, they reinforce the ancestral state of rockfish to be long lived. The intersection of these models provides previously unappreciated yet evolutionary entrenched mechanisms associating with increased life span in diverse vertebrate lineages.

We have capitalized on variation in clades exhibiting exceptional longevity through convergence analyses and translated those results with functional gene sets to identify variation associating with longevity in human populations. Age is the greatest risk factor for the diseases that plague modern society—cancer, Alzheimer’s, heart disease, etc.—and medical research has traditionally focused on ameliorating these pathologies individually. These exceptional fish have already arrived at solutions to these aging problems, while others even have age-related improvements in health (*90*). The specific targets identified here, and their unique variations in both humans and rockfish, are auspicious targets for future interventions to delay, ameliorate, or even prevent aging and age-related diseases in humans.

## METHODS

### Tissue sampling

Rockfish tissue samples were obtained from those available in collections maintained by Oregon State University and the Burke Museum (curt. L. Torneborne). Sea Robin tissues were provided by Department of Marine Resources, Marine Biology Laboratories, Woods Hole. Sample identity and source can be found in table S1. Initially identified by expert naturalists, species were confirmed by polymerase chain reaction amplification and Sanger sequencing of mitochondrial DNA–encoded cytochrome B and cytochrome C oxidase 1. All species used in this study are not listed by the Convention on International Trade in Endangered Species of Wild Fauna and Flora. Collections adhered to the principles of the Nagoya protocol. Genomic datasets and diversity information are supplied without access and benefit sharing restrictions.

### Capture design, sequencing, and assembly

#### Targeted sequence capture design

The sequence capture design targets protein-coding exons and a set of conserved noncoding elements (CNEs), miRNA hairpins, and ultraconservative noncoding elements (UCNEs). As many of the available perciform genomes were fragmented and poorly annotated at the time of sequence capture design, we generated a list of conserved protein coding and CNE regions from the genomes of the three-spined stickleback *Gasterosteus aculeatus* (BROAD S1), the Japanese medaka (*Oryzias latipes*, MEDAKA1), and green spotted puffer *Tetraodon nigroviridis* (TETRAODON 8.0). Protein-coding exons were extracted from Ensembl BioMart (*91*). CNEs were defined from the constrained regions in the Ensembl compara 11-way teleost alignment (Ensembl release-91) (*92*). We removed CNEs <50 base pairs (bp) in length to facilitate space in the capture design. miRNA hairpins were extracted from miRbase and ultraconservative elements (UCNEs) from UCNEbase (*93*, *94*). These elements were identified within each reference genome using BLASTN (ncbi-blast-2.2.30+) or through direct annotations where available. miRNA hairpins were padded to be at least 100 bp. To verify these sites as noncoding, we eliminated CNEs, miRNAs, and UCNEs that overlapped coding exons using Bedtools (v2.26.0) intersectBed (*95*). In the event CNEs were also defined as miRNAs or UCNEs, we prioritized miRNA and UCNE annotations.

We aimed to design a series of oligonucleotide capture baits that could efficiently enrich DNA sequencing libraries across Perciformes, a large order of more than 2200 species (*96*). We used BLASTN (ncbi-blast-2.2.30+) to identify each targeted element within multiple perciform genome assemblies. The majority of capture baits were designed against the genome of the Chabot de Rhénanie *Cottus rhenanus* (ASM145555v1). To account for the possibility that specific genetic regions may be absent or highly divergent in this sculpin genome but conserved in the broader Perciformes order, we iteratively designed capture baits from the genomes of the shorthorn sculpin *Myoxocephalus scorpius* (ASM90031295v1) (*97*), the sablefish *Anoplopoma fimbria* (AnoFim1.0) (*98*), the golden redfish *Sebastes norvegicus* (ASM90030265v1) (*97*), the flag rockfish *Sebastes rubrivinctus* (SRub1.0), the rougheye rockfish *S. aleutianus* (ASM191080v2), the European perch *Perca fluviatilis* (ASM90030264v1) (*97*), and the three-spined stickleback *G. aculeatus* (BROAD S1). As an example, we included capture baits from the *M. scorpius* genome if the targeted elements were either not identified (coverage of <70% or a BLASTN *E* value > 0.001) and/or had <85% identity to the orthologous element within the *C. rhenanus* genome. This process is then repeated such that elements from *A. fimbria* are compared to both the *M. scorpius* and *C. rhenanus* targets. As a result of this process, there will be oligonucleotide capture baits of at least 85% identity to each targeted sequence for every perciform genome used in the capture design. This multispecies “phylochip” design enables usage of these baits to sequence large numbers of distantly related perciform fish species.

SeqCap EZ Developer (catalog no. 06471684001) capture oligos were designed in collaboration with the NimbleGen design team to standardize oligo annealing temperature, to reduce probe redundancy, and to remove low-complexity DNA regions. The capture design contained sequence from 492,506 regions (81,493,221 total bp) across all eight perciform reference genomes. Accounting for probe redundancy between the perciform reference genomes, the final capture design comprised 407,084 distinct elements, including 285,872 protein coding exons, 118,406 conserved noncoding elements, 298 miRNAs, and 2508 UCNEs (fig. S1).

#### Sequencing library preparation

DNA was extracted from multiple individuals of each species using the Qiagen DNeasy Blood & Tissue Kit (catalog no. 69504). For each species, equal quantities of DNA were pooled from each individual before library preparation and sequencing. The DNA pools were diluted in a shearing buffer [10 mM tris and 0.1 mM EDTA (pH 8.3)] and were mechanically sheared to an average size of 200 bp in a Covaris E220 ultrasonicator (duty cycle, 10%; intensity, 5; cycles/burst, 200; time, 400 s; temperature, 8°C). Barcoded sequencing libraries were generated using the KAPA HyperPrep Kit (Roche, no. 07137923001) following the NimbleGen SeqCap EZ Library protocol (version 4.3) and using dual solid-phase reversible immobilization size selection to generate libraries of 200 to 450 bp. The libraries were hybridized to the capture baits according to the SeqCap EZ Library protocol. To increase allowance for mismatches between libraries and baits, the libraries were hybridized to the capture baits and washed at a reduced stringency of 45°C instead of the manufacturer recommended temperature of 47°C. The SeqCap EZ Developer Reagent was used (catalog no. 06684335001) in place of Human CotI DNA during hybridization. Multiple barcoded libraries were then pooled for 100-bp single-end sequencing with an Illumina HiSeq 2500.

#### Reference contig assembly

We used the Phylomapping de novo assembly pipeline to generate reference contigs for each species (*99*). Briefly, we removed duplicate reads and masked low-quality bases with the FASTX toolkit (http://hannonlab.cshl.edu/fastx_toolkit). Library adaptor sequences were removed using Trimmomatic v.0.36 (*100*). Sequencing reads were binned by homology to target regions in the *G. aculeatus*, *T. nigroviridis*, and *O. latipes* reference genomes using BLASTN and dc-megaBLAST. The binned sequencing reads were assembled into contigs de novo using CAP3 (*101*) and UCLUST (*102*). To recruit previously unmapped reads to the analysis, all sequencing reads were aligned back to the assembled contigs using NextGenMap (*103*). All reads were reassembled using CAP3 in a second round of de novo assembly. If multiple contigs are assembled for any given element (for example, exon and CNE), then the multiple contigs were then merged if they overlapped and had >95% identity. This pipeline results in the creation of consensus contig sequence(s) for each target region. Contigs were automatically annotated according to the homologous element within the reference genome that was identified using BLAST and subsequently used to scaffold and refine contig assembly. See Daane *et al*. (*99*) for details on these methods and settings.

#### Identification of orthologs

Contigs from each species are considered orthologs if they are assembled based on the same targeted element. However, if multiple contigs are assembled for a given targeted element, then we assigned orthology using a gene-tree species-tree reconciliation approach. Contigs for all species were aligned using MAFFT v7.313 (*104*), and a maximum likelihood tree was generated using IQTree v1.7beta2 (*105*). We then used Notung-2.9 (*106*) to perform gene tree reconciliation and to infer patterns of gene loss and duplication. The total number of duplication and loss events across the phylogeny as estimated by Notung was then compared to a scenario where all copy number variation is species specific. The most parsimonious scenario with the fewest total gain/loss events was selected. Using this approach, orthologs can be accurately paired as long as there ≥4 to 6% variation between paralogous sequences, which coincides with thresholds of variation necessary to distinguish copy number variation during contig assembly. See Daane *et al*. (*99*) for details on these methods and settings.

#### Read coverage and depth of targeted regions

The target regions (exons, CNEs, etc.) were originally defined from the *G. aculeatus*, *T. nigroviridis*, and *O. latipes* reference genomes. To estimate sequencing coverage of these targeted regions in our assembly, we lifted our assembled contig read alignment data to the orthologous region within each reference genome. Sequencing reads were aligned to the assembled contigs using NextGenMap v0.5.5 (*103*). The assembled contigs were then pairwise aligned to the target regions within the reference genome using Biopython v.1.70. Data from this pairwise alignment, including indels, were used to map the aligned sequencing reads from the assembled contigs to the reference genome. Alignments were converted from SAM to BAM format and indexed using SAMtools (v1.9) (*107*) and were visually inspected for accuracy in the Integrative Genome Viewer (*108*). Coverage is defined as the proportion of targeted bases overlapping at least one sequencing read. Coverage and depth was calculated with Bedtools v2.23.0 coverageBed (*95*). See Daane *et al*. (*99*) for details on these methods and settings.

#### Multiple sequence alignment and gene reconstruction

Orthologous sequences were aligned using MAFFT v 7.313 (*104*). If the MAFFT alignment predicted an indel within coding sequences, then we realigned these exons as codon alignments using the frameshift-aware multiple sequence alignment software MACSE v2.03 (*109*). Individual exons were reconstructed into full gene sequences. Single-copy exons were concatenated in gene order as found in the *G. aculeatus* (three-spined stickleback; BROAD S1), *T. nigroviridis* (green spotted pufferfish; TETRAODON 8.0), or *O. latipes* (Japanese medaka, MEDAKA1) reference genomes. See Daane *et al*. (*99*) for details on these methods and settings. For each rockfish, we reconstructed a total of 27,645 gene sequences.

### Analyses and statistics

#### Species tree construction

The already aligned coding sequences with greater than 90% coverage in every sampled species, and longer than 2000 bases, were concatenated. The resulting 6,530,274 bases were analyzed with IQTree’s substitution model finder (*27*), identifying GY+F+R3 and then generating the rockfish phylogeny with UFBoot (*28*) with greater than 99% support at every node. Individual gene trees for rate analysis used the same model and were fixed to the species tree topology.

#### Phylogenetic statistics

The pgls and pglmm calculations were performed in R with the nlme (*110*) and ape packages (*111*). *R*^2^_pred_ was calculated using the rr2 package (*26*).

#### Relative rate analysis

Convergent rate analysis was performed with TRACCER (*31*) on the 23 rockfish species and sea robin outgroup. *Sebastes borealis*, *aleutianus*, *crameri*, *alutus*, *ruberrimus*, *melanostomus*, *diploproa*, *pinniger*, *maliger*, *melanops*, *rufus*, *babcocki*, and *helvomaculatus* were flagged as long lived, while *S. emphaeus*, *glaucus*, *seminictus*, *rastrelliger*, *auriculatus*, *jordani*, *hopkinsi*, *rubrivinctus*, *umbrosus*, *chlorostictus*, and *P. carolinus* were flagged as short lived. The control selection was chosen to recapitulate similar phylogenetic relationships while inverting half of the assignments, flagging *diploproa*, *maliger*, *carolinus*, *glaucus*, *borealis*, *umbrosus*, *hopkinsi*, *rubrivinctus*, *rufus*, *alutus*, *seminictus*, *auriculatus*, and *pinniger* as long lived and *chlorostictus*, *helvomaculatus*, *aleutianus*, *babcocki*, *melanostomus*, *rastrelliger*, *ruberrimus*, *jordani*, *emphaeus*, *crameri*, and *melanops* as short lived. Gene trees were discarded if they had fewer than six long-lived lineages, or five short-lived lineages, or 12 total, as the rates of the remaining representatives were unlikely to be broadly informative for the trait. The ranking transformation was used for both scoring and phylogenetic distance scaling. FDRs (*q* values) are calculated as the expected number of hits at that significance divided by the number of actual hits at that significance or better (*112*).

#### Detecting selective pressures

The coding sequences for each gene in the flavonoid metabolism gene set were concatenated together, for each lineage, while removing codons missing coverage in any rockfish lineages. HyPhy (v2.5.41) RELAX and aBSREL were run with standard settings while additionally allowing synonymous rate variation. The short-lived terminal rockfish branches were flagged as the foreground and compared to the background rates of all other branches on the tree. Uncorrected *P* values were reported for aBSREL; as we were testing the hypothesis that the short lived would share selective pressures, not all branches were individually of interest. This was repeated for the glycogen biosynthesis gene set.

#### Gene set enrichment

GO annotations were harvested from Ensembl by combining the annotations across orthologs of well-characterized genomes, including human, mice, zebrafish, stickleback, and medaka. Terms with less than three members, or more than one hundred, were discarded as being uninformative or misleading in the context of set enrichment. Gene set enrichment was calculated with the SUMSTAT approach, which has demonstrated power, flexibility, and simplicity over competing gene set enrichment analyses (*113*–*115*). SUMSTAT was performed with log-transformed *P* values from each analysis, with an additional square root transformation to undermine outliers. FDRs are calculated as above. These methods were applied with both direct and indirect (those that are propagated through the GO hierarchy) annotations. Direct annotations were used when trying to minimize redundancy between terms. Indirect annotations were used when feeding into network analyses, ensuring the broader scopes are included, while using only the highest-ranked (*P* < 0.1) genes in each significant term (*q* < 0.1).

#### Genetics of human longevity

Orthologs from fish to humans were taken directly from Ensembl when available (ensembl.org). Those that did not have an annotated ortholog were mapped using pBLAST against the human proteome and confirmed if their best hit had an *E* value less than 1 × 10^−30^ and percent identity greater than 40. The remaining fish genes were dropped. The orthologous human genes were used with data from GWAS for human longevity (*21*). MAGMA (*116*) was used to assign *P* values to genes based on the lowest *P* value for a genetic variant within or around a gene coordinates, including 50 kb up- and downstream. The association data of the genetic variants used to assign the *P* values was extracted from the 90th and 99th survival percentile analyses summary results from Deelen *et al.* (*21*). Gene set enrichment analysis was performed using the GO terms coming from the rockfish with the human orthologous genes using the “gene-model snp-wise = top” setting in MAGMA. We decided to use this setting because we expect that only a small proportion of SNPs in each gene will show an association with longevity, and, according to the manual (*116*), this is the most sensitive setting in these situations.

## Supplementary Materials

### This PDF file includes:

Figs. S1 and S2 References

## Notes

View/request a protocol for this paper from *Bio-protocol*.
