# Supplementary material for: Convergent genomics of longevity in rockfishes highlights the genetics of human life span variation
Source: Sci Adv. 2023 Jan 11;9(2):eadd2743. doi: 10.1126/sciadv.add2743 (PMC9833670; doi:10.1126/sciadv.add2743)
Supplement: Supplementary file 1 — Figs. S1 and S2 References [file sciadv.add2743_sm.pdf]

Science Advances
AAAS

Supplementary Materials for

Convergent genomics of longevity in rockfishes highlights the genetics of human life span variation

Stephen Treaster *et al.*

Corresponding author: Stephen Treaster, stephen_treaster@hms.harvard.edu;
Matthew P. Harris, harris@genetics.med.harvard.edu

Sci. Adv. **9**, eadd2743 (2023)
DOI: 10.1126/sciadv.add2743

The PDF file includes:

Figs. S1 and S2
Legends for tables S1 to S7
References

Other Supplementary Material for this manuscript includes the following:

Tables S1 to S7

Supplementary Materials

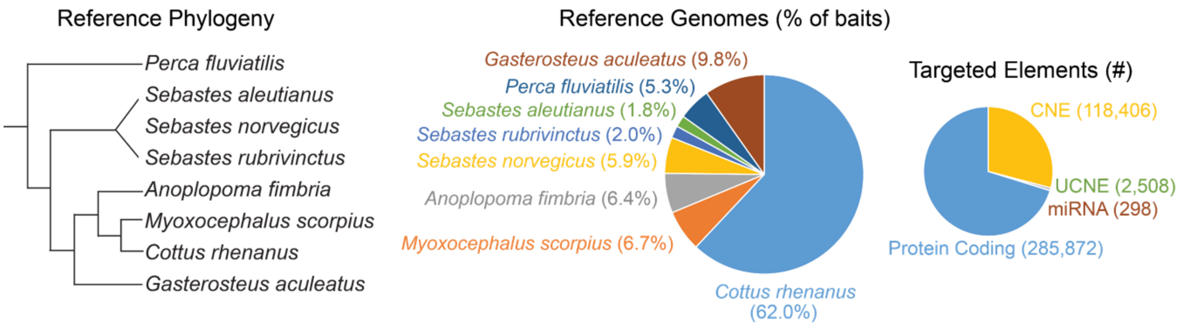

Supplemental Figure 1. Perciforme Capture Design. The sequence capture design targets protein-coding exons as well as a set of conserved non-protein coding elements (CNEs), miRNA hairpins, and ultraconservative non-coding elements (UCNEs). As many of the available perciform genomes were fragmented and poorly annotated at the time of sequence capture design, we generated a list of conserved protein coding and CNE regions from the genomes of the three-spined stickleback (*Gasterosteus aculeatus*), the Japanese medaka (*Oryzias latipes*) and green spotted puffer (*Tetraodon nigroviridis*). A diversity of reference perciformes were then iteratively mined for these regions if their orthologous sequence was less than 85% identical to the baits already in the capture. As a result of this process, there will be oligonucleotide capture baits of at least 85% identity to each targeted sequence for every perciform genome used in the capture design. This multi-species ‘phylochip’ design enables usage of these baits to sequence large numbers of distantly-related perciform fish species.

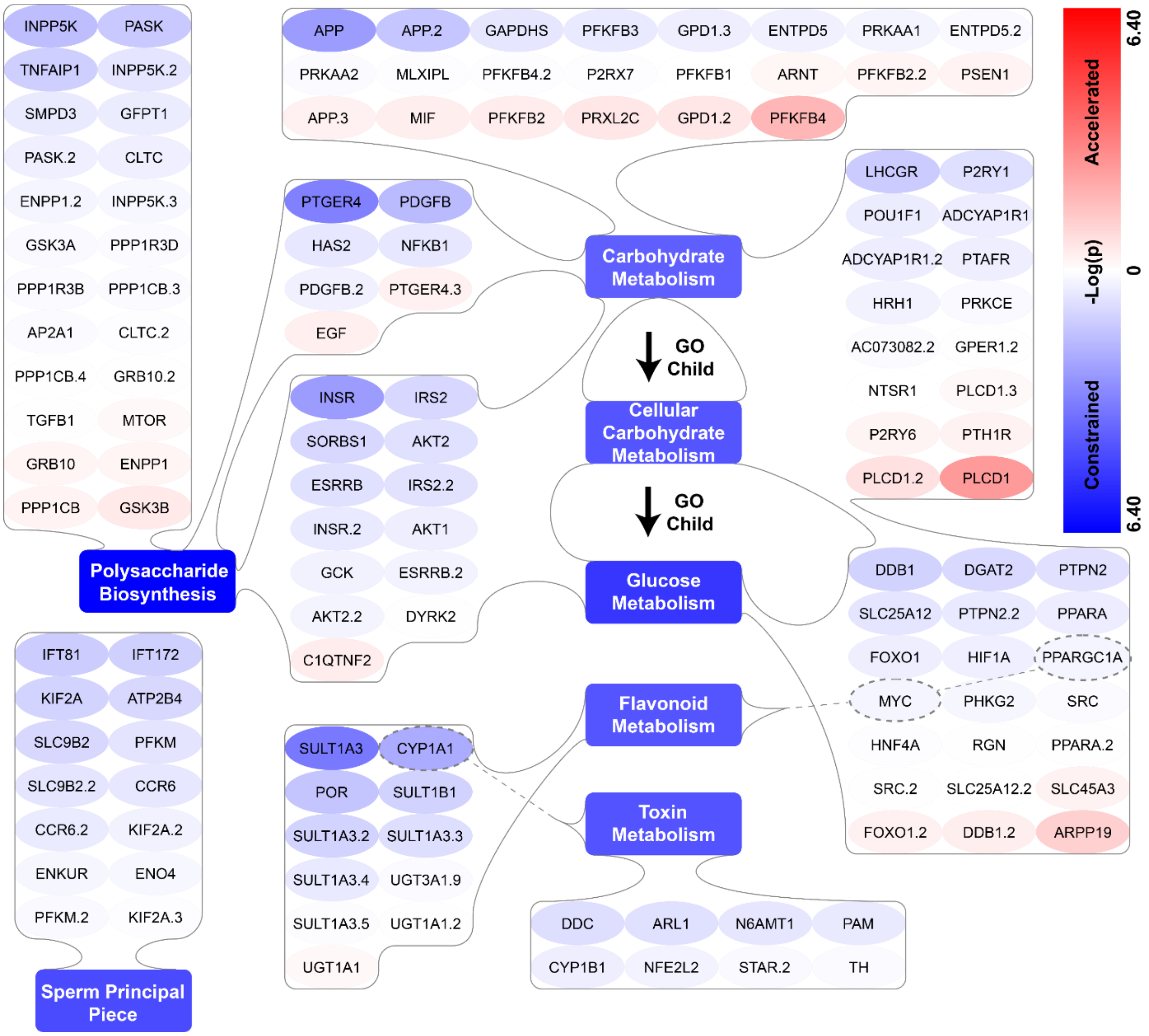

Supplemental Figure 2. Expanded GO Terms with significant relative evolutionary rate shifts in Rockfish longevity. GO Enrichment with propagated (indirect) annotations. Gene groupings demonstrate the degrees of interdependence between the various enriched functional groups. Genes in these functional groups are consistently and significantly under constrained (blue) evolutionary rates with extended longevity (relative to short-lived lineages), while accelerated genes (red) are rare. These trends yield even greater signals in the functional groups than in the individual genes. The genes driving these signals are sorted to the top left of each grouping, and include hallmark aging candidates, such as INSR, APP, and LHCGR. Due to the complexities of mapping fish paralogues, some genes are appended additional identifiers.

Supplementary Tables

Table 1: Species and Sampling

Table 2: Coverage

Table 3: TRACCER Convergent Rate

Table 4: Rockfish Indirect GO Gene Set Enrichment

Table 5: Rockfish Direct GO Gene Set Enrichment

Table 6: CHARGE Human Gene Set Enrichment

Table 7: CHARGE Human Gene Set Enrichment Genes

REFERENCES AND NOTES

1. A. A. Cohen, Aging across the tree of life: The importance of a comparative perspective for the use of animal models in aging. *Biochim. Biophys. Acta Mol. Basis Dis.* **1864**, 2680–2689 (2018).

2. X. Tian, A. Seluanov, V. Gorbunova, Molecular mechanisms determining lifespan in short- and long-lived species. *Trends Endocrinol. Metab.* **28**, 722–734 (2017).

3. H. Liang, E. J. Masoro, J. F. Nelson, R. Strong, C. A. McMahan, A. Richardson, Genetic mouse models of extended lifespan. *Exp. Gerontol.* **38**, 1353–1364 (2003).

4. I. D. Ridgway, C. A. Richardson, S. N. Austad, Maximum shell size, growth rate, and maturation age correlate with longevity in bivalve molluscs. *J. Gerontol. A Biol. Sci. Med. Sci.* **66**, 183–190 (2010).

5. J. Nielsen, R. B. Hedeholm, J. Heinemeier, P. G. Bushnell, J. S. Christiansen, J. Olsen, C. B. Ramsey, R. W. Brill, M. Simon, K. F. Steffensen, J. F. Steffensen, Eye lens radiocarbon reveals centuries of longevity in the Greenland shark (*Somniosus microcephalus*). *Science* **353**, 702–704 (2016).

6. M. Keane, J. Semeiks, A. E. Webb, Y. I. Li, V. Quesada, T. Craig, L. B. Madsen, S. van Dam, D. Brawand, P. I. Marques, P. Michalak, L. Kang, J. Bhak, H.S. Yim, N. V. Grishin, N. H. Nielsen, M. P. Heide-Jørgensen, E. M. Oziolor, C. W. Matson, G. M. Church, G. W. Stuart, J. C. Patton, J. C. George, R. Suydam, K. Larsen, C. López-Otín, M. J. O'Connell, J. W. Bickham, B. Thomsen, J. P. de Magalhães, Insights into the evolution of longevity from the bowhead whale genome. *Cell Rep.* **10**, 112–122 (2015).

7. K. M. Munk, Maximum ages of groundfishes in waters off Alaska and British Columbia and considerations of age determination. *Alsk. Fish. Res. Bull.* **8**, 12–21 (2001).

8. C. R. Kastelle, D. K. Kimura, S. R. Jay. Using 210Pb/226Ra disequilibrium to validate conventional ages in Scorpaenids (genera *Sebastes* and *Sebastolobus*). *Fish. Res.* **46**, 299–312 (2000).

9. E. A. Kikis, T. Gidalevitz, R. I. Morimoto, Protein homeostasis in models of aging and age-related conformational disease. *Adv. Exp. Med. Biol.* **694**, 138–159 (2010).

10. H. Koga, S. Kaushik, A. M. Cuervo, Protein homeostasis and aging: The importance of exquisite quality control. *Ageing Res. Rev.* **10**, 205–215 (2011).

11. J. Heras, A. Aguilar, Comparative transcriptomics reveals patterns of adaptive evolution associated with depth and age within marine rockfishes (*Sebastes*). *J. Hered.* **110**, 340–350 (2019).

12. S. R. R. Kolora, G. L. Owens, J. M. Vazquez, A. Stubbs, K. Chatla, C. Jainese, K. Seeto, M. McCrea, M. W. Sandel, J. A. Vianna, K. Maslenikov, D. Bachtrog, J. W. Orr, M. Love, P. H. Sudmant, Origins and evolution of extreme life span in Pacific Ocean rockfishes. *Science* **374**, 842–847 (2021).

13. J. R. Hyde, R. D. Vetter, The origin, evolution, and diversification of rockfishes of the genus *Sebastes* (Cuvier). *Mol. Phylogenet. Evol.* **44**, 790–811 (2007).

14. X. Hua, P. Cowman, D. Warren, L. Bromham, Longevity is linked to mitochondrial mutation rates in rockfish: A test using poisson regression. *Mol. Biol. Evol.* **32**, 2633–2645 (2015).

15. S. N. Austad, K. E. Fischer, Mammalian aging, metabolism, and ecology: Evidence from the bats and marsupials. *J. Gerontol.* **46**, B47–B53 (1991).

16. E. T. Chan, G. T. Quon, G. Chua, T. Babak, M. Trochesset, R. A. Zirngibl, J. Aubin, M. J. Ratcliffe, A. Wilde, M. Brudno, Q. D. Morris, T. R. Hughes, Conservation of core gene expression in vertebrate tissues. *J. Biol.* **8**, 33 (2009).

17. K. Lindblad-Toh, M. Garber, O. Zuk, M. F. Lin, B. J. Parker, S. Washietl, P. Kheradpour, J. Ernst, G. Jordan, E. Mauceli, L. D. Ward, C. B. Lowe, A. K. Holloway, M. Clamp, S. Gnerre, J. Alföldi, K. Beal, J. Chang, H. Clawson, J. Cuff, F. di Palma, S. Fitzgerald, P. Flicek, M. Guttman, M. J. Hubisz, D.B. Jaffe, I. Jungreis, W. J. Kent, D. Kostka, M. Lara, A. L. Martins, T. Massingham, I. Moltke, B. J. Raney, M.D. Rasmussen, J. Robinson, A. Stark, A. J. Vilella, J. Wen, X. Xie, M.C. Zody; Broad Institute Sequencing Platform and Whole Genome

Assembly Team, J. Baldwin, T. Bloom, C.W. Chin, D. Heiman, R. Nicol, C. Nusbaum, S. Young, J. Wilkinson, K. C. Worley, C. L. Kovar, D. M. Muzny, R. A. Gibbs; Baylor College of Medicine Human Genome Sequencing Center Sequencing Team, A. Cree, H. H. Dihn, G. Fowler, S. Jhangiani, V. Joshi, S. Lee, L. R. Lewis, L. V. Nazareth, G. Okwuonu, J. Santibanez, W. C. Warren, E. R. Mardis, G. M. Weinstock, R. K. Wilson; Genome Institute at Washington University, K. Delehaunty, D. Dooling, C. Fronik, L. Fulton, B. Fulton, T. Graves, P. Minx, E. Sodergren, E. Birney, E. H. Margulies, J. Herrero, E. D. Green, D. Haussler, A. Siepel, N. Goldman, K. S. Pollard, J. S. Pedersen, E. S. Lander, M. Kellis, A high-resolution map of human evolutionary constraint using 29 mammals. *Nature* **478**, 476–482 (2011).

18. A. Kowalczyk, R. Partha, N. L. Clark, M. Chikina, Pan-mammalian analysis of molecular constraints underlying extended lifespan. *eLife* **9**, e51089 (2020).

19. B. Mayne, O. Berry, C. Davies, J. Farley, S. Jarman, A genomic predictor of lifespan in vertebrates. *Sci. Rep.* **9**, 17866 (2019).

20. S. Treaster, D. Karasik, M. P. Harris, Footprints in the sand: Deep taxonomic comparisons in vertebrate genomics to unveil the genetic programs of human longevity. *Front. Genet.* **12**, 678073 (2021).

21. J. Deelen, D. S. Evans, D. E. Arking, N. Tesi, M. Nygaard, X. Liu, M. K. Wojczynski, M. L. Biggs, A. van der Spek, G. Atzmon, E. B. Ware, C. Sarnowski, A. V. Smith, I. Seppälä, H. J. Cordell, J. Dose, N. Amin, A. M. Arnold, K. L. Ayers, N. Barzilai, E. J. Becker, M. Beekman, H. Blanché, K. Christensen, L. Christiansen, J. C. Collerton, S. Cubaynes, S. R. Cummings, K. Davies, B. Debrabant, J.F. Deleuze, R. Duncan, J. D. Faul, C. Franceschi, P. Galan, V. Gudnason, T. B. Harris, M. Huisman, M. A. Hurme, C. Jagger, I. Jansen, M. Jylhä, M. Kähönen, D. Karasik, S. L. R. Kardia, A. Kingston, T. B. L. Kirkwood, L. J. Launer, T. Lehtimäki, W. Lieb, L.P. Lyytikäinen, C. Martin-Ruiz, J. Min, A. Nebel, A. B. Newman, C. Nie, E. A. Nohr, E. S. Orwoll, T. T. Perls, M. A. Province, B. M. Psaty, O. T. Raitakari, M. J. T. Reinders, J.M. Robine, J. I. Rotter, P. Sebastiani, J. Smith, T. I. A. Sørensen, K. D. Taylor, A. G. Uitterlinden, W. van der Flier, S. J. van der Lee, C. M. van Duijn, D. van Heemst, J. W.

Vaupel, D. Weir, K. Ye, Y. Zeng, W. Zheng, H. Holstege, D. P. Kiel, K. L. Lunetta, P. E. Slagboom, J. M. Murabito, A meta-analysis of genome-wide association studies identifies multiple longevity genes. *Nat. Commun.* **10**, 3669 (2019).

22. P. Gayral, J. Melo-Ferreira, S. Glémin, N. Bierne, M. Carneiro, B. Nabholz, J. M. Lourenco, P. C. Alves, M. Ballenghien, N. Faivre, K. Belkhir, V. Cahais, E. Loire, A. Bernard, N. Galtier, Reference-free population genomics from next-generation transcriptome data and the vertebrate-invertebrate gap. *PLOS Genet.* **9**, e1003457 (2013).

23. E. B. Kim, X. Fang, A. A. Fushan, Z. Huang, A. V. Lobanov, L. Han, S. M. Marino, X. Sun, A. A. Turanov, P. Yang, S. H. Yim, X. Zhao, M. V. Kasaikina, N. Stoletzki, C. Peng, P. Polak, Z. Xiong, A. Kiezun, Y. Zhu, Y. Chen, G. V. Kryukov, Q. Zhang, L. Peshkin, L. Yang, R. T. Bronson, R. Buffenstein, B. Wang, C. Han, Q. Li, L. Chen, W. Zhao, S. R. Sunyaev, T. J. Park, G. Zhang, J. Wang, V. N. Gladyshev, Genome sequencing reveals insights into physiology and longevity of the naked mole rat. *Nature* **479**, 223–227 (2011).

24. M. Wirthlin, N. C. B. Lima, R. L. M. Guedes, A. E. R. Soares, L. G. P. Almeida, N. P. Cavaleiro, G. L. de Morais, A. V. Chaves, J. T. Howard, M. de Melo Teixeira, P. N. Schneider, F. R. Santos, M. C. Schatz, M. S. Felipe, C. Y. Miyaki, A. Aleixo, M. P. C. Schneider, E. D. Jarvis, A. T. R. Vasconcelos, F. Prosdocimi, C. V. Mello, Parrot genomes and the evolution of heightened longevity and cognition. *Curr. Biol.* **28**, 4001–4008.e7 (2018).

25. J. M. Daane, N. Rohner, P. Konstantinidis, S. Djuranovic, M. P. Harris, Parallelism and epistasis in skeletal evolution identified through use of phylogenomic mapping strategies. *Mol. Biol. Evol.* **33**, 162–173 (2016).

26. A. R. Ives, R^2s for correlated data: Phylogenetic models, LMMs, and GLMMs. *Syst. Biol.* **68**, 234–251 (2019).

27. S. Kalyaanamoorthy, B. Q. Minh, T. K. F. Wong, A. von Haeseler, L. S. Jermiin, ModelFinder: Fast model selection for accurate phylogenetic estimates. *Nat. Methods* **14**, 587–589 (2017).

28. D. T. Hoang, O. Chernomor, A. von Haeseler, B. Q. Minh, L. S. Vinh, UFBoot2: Improving the ultrafast bootstrap approximation. *Mol. Biol. Evol.* **35**, 518–522 (2018).

29. G. W. C. Thomas, R. J. Wang, A. Puri, R. A. Harris, M. Raveendran, D. S. T. Hughes, S. C. Murali, L. E. Williams, H. Doddapaneni, D. M. Muzny, R. A. Gibbs, C. R. Abee, M. R. Galinski, K. C. Worley, J. Rogers, P. Radivojac, M. W. Hahn, Reproductive longevity predicts mutation rates in primates. *Curr. Biol.* **28**, 3193–3197.e5 (2018).

30. M. Lynch, The cellular, developmental and population-genetic determinants of mutation-rate evolution. *Genetics* **180**, 933–943 (2008).

31. S. Treaster, J. M. Daane, M. P. Harris, Refining convergent rate analysis with topology in mammalian longevity and marine transitions. *Mol. Biol. Evol.* **38**, 5190–5203 (2021).

32. M. D. Smith, J. O. Wertheim, S. Weaver, B. Murrell, K. Scheffler, S. L. Kosakovsky Pond, Less is more: An adaptive branch-site random effects model for efficient detection of episodic diversifying selection. *Mol. Biol. Evol.* **32**, 1342–1353 (2015).

33. J. O. Wertheim, B. Murrell, M. D. Smith, S. L. K. Pond, K. Scheffler, RELAX: Detecting relaxed selection in a phylogenetic framework. *Mol. Biol. Evol.* **32**, 820–832 (2015).

34. C. J. Kenyon, The genetics of ageing. *Nature* **464**, 504–512 (2010).

35. P. P. Singh, B. A. Demmitt, R. D. Nath, A. Brunet, The genetics of aging: A vertebrate perspective. *Cell* **177**, 200–220 (2019).

36. R. Cui, T. Medeiros, D. Willemsen, L. N. M. Iasi, G. E. Collier, M. Graef, M. Reichard, D. R. Valenzano, Relaxed selection limits lifespan by increasing mutation load. *Cell* **178**, 385–399.e20 (2019).

37. Y. Wang, J. Yang, T. Hong, X. Chen, L. Cui, SIRT2: Controversy and multiple roles in disease and physiology. *Ageing Res. Rev.* **55**, 100961 (2019).

38. U. C. Müller, T. Deller, M. Korte, Not just amyloid: Physiological functions of the amyloid precursor protein family. *Nat. Rev. Neurosci.* **18**, 281–298 (2017).

39. P. Porayette, M. J. Gallego, M. M. Kaltcheva, S. V. Meethal, C. S. Atwood, Amyloid-beta precursor protein expression and modulation in human embryonic stem cells: A novel role for human chorionic gonadotropin. *Biochem. Biophys. Res. Commun.* **364**, 522–527 (2007).

40. P. Sebastiani, N. Solovieff, A. T. DeWan, K. M. Walsh, A. Puca, S. W. Hartley, E. Melista, S. Andersen, D. A. Dworkis, J. B. Wilk, R. H. Myers, M. H. Steinberg, M. Montano, C. T. Baldwin, J. Hoh, T. T. Perls, Genetic signatures of exceptional longevity in humans. *PLOS ONE* **7**, e29848 (2012).

41. P. Sebastiani, H. Bae, F. X. Sun, S. L. Andersen, E. W. Daw, A. Malovini, T. Kojima, N. Hirose, N. Schupf, A. Puca, T. T. Perls, Meta-analysis of genetic variants associated with human exceptional longevity. *Aging* **5**, 653–661 (2013).

42. A. Augert, C. Payré, Y. de Launoit, J. Gil, G. Lambeau, D. Bernard, The M-type receptor PLA2R regulates senescence through the p53 pathway. *EMBO Rep.* **10**, 271–277 (2009).

43. D. Bernard, D. Vindrieux, PLA2R1: Expression and function in cancer. *Biochim. Biophys. Acta Rev. Cancer* **1846**, 40–44 (2014).

44. A. Griveau, C. Wiel, B. le Calvé, D. V. Ziegler, S. Djebali, M. Warnier, N. Martin, J. Marvel, D. Vindrieux, M. O. Bergo, D. Bernard, Targeting the phospholipase A2 receptor ameliorates premature aging phenotypes. *Aging Cell* **17**, e12835 (2018).

45. D. Vindrieux, A. Augert, C. A. Girard, D. Gitenay, H. Lallet-Daher, C. Wiel, B. le Calvé, B. Gras, M. Ferrand, S. Verbeke, Y. de Launoit, X. Leroy, A. Puisieux, S. Aubert, M. Perrais, M. Gelb, H. Simonnet, G. Lambeau, D. Bernard, PLA2R1 mediates tumor suppression by activating JAK2. *Cancer Res.* **73**, 6334–6345 (2013).

46. S. Mostafavi, D. Ray, D. Warde-Farley, C. Grouios, Q. Morris, GeneMANIA: A real-time multiple association network integration algorithm for predicting gene function. *Genome Biol.* **9**, S4 (2008).

47. M. Depczynski, D. R. Bellwood, Shortest recorded vertebrate lifespan found in a coral reef fish. *Curr. Biol.* **15**, R288–R289 (2005).

48. A. A. Maklakov, S. Immler, The expensive germline and the evolution of ageing. *Curr. Biol.* **26**, R577–R586 (2016).

49. B. R. Holland, S. Ketelaar-Jones, A. R. O'Mara, M. D. Woodhams, G. J. Jordan, Accuracy of ancestral state reconstruction for non-neutral traits. *Sci. Rep.* **10**, 7644 (2020).

50. A. Kowalczyk, W. K. Meyer, R. Partha, W. Mao, N. L. Clark, M. Chikina, RERconverge: An R package for associating evolutionary rates with convergent traits. *Bioinformatics* **35**, 4815–4817 (2019).

51. J. B. Dorman, B. Albinder, T. Shroyer, C. Kenyon, The age-1 and daf-2 genes function in a common pathway to control the lifespan of *Caenorhabditis elegans*. *Genetics* **141**, 1399–1406 (1995).

52. C. Kenyon, J. Chang, E. Gensch, A. Rudner, R. Tabtiang, A *C. elegans* mutant that lives twice as long as wild type. *Nature* **366**, 461–464 (1993).

53. I. Gusarov, B. Pani, L. Gautier, O. Smolentseva, S. Eremina, I. Shamovsky, O. Katkova-Zhukotskaya, A. Mironov, E. Nudler, Glycogen controls *Caenorhabditis elegans* lifespan and resistance to oxidative stress. *Nat. Commun.* **19**, 15868 (2017).

54. M. Holzenberger, J. Dupont, B. Ducos, P. Leneuve, A. Géloën, P. C. Even, P. Cervera, Y. le Bouc, IGF-1 receptor regulates lifespan and resistance to oxidative stress in mice. *Nature* **421**, 182–187 (2003).

55. J. Deelen, H. W. Uh, R. Monajemi, D. van Heemst, P. E. Thijssen, S. Böhringer, E. B. van den Akker, A. J. M. de Craen, F. Rivadeneira, A. G. Uitterlinden, R. G. J. Westendorp, J. J. Goeman, P. E. Slagboom, J. J. Houwing-Duistermaat, M. Beekman, Gene set analysis of GWAS data for human longevity highlights the relevance of the insulin/IGF-1 signaling and telomere maintenance pathways. *Age* **35**, 235–249 (2013).

56. F. Flachsbart, J. Dose, L. Gentschew, C. Geismann, A. Caliebe, C. Knecht, M. Nygaard, N. Badarinarayan, A. ElSharawy, S. May, A. Luzius, G. G. Torres, M. Jentzsch, M. Forster, R. Häsler, K. Pallauf, W. Lieb, C. Derbois, P. Galan, D. Drichel, A. Arlt, A. Till, B. Krause-Kyora, G. Rimbach, H. Blanché, J. F. Deleuze, L. Christiansen, K. Christensen, M. Nothnagel, P. Rosenstiel, S. Schreiber, A. Franke, S. Sebens, A. Nebel, Identification and characterization of two functional variants in the human longevity gene FOXO3. *Nat. Commun.* **8**, 2063 (2017).

57. V. Grossi, G. Forte, P. Sanese, A. Peserico, T. Tezil, M. Lepore Signorile, C. Fasano, R. Lovaglio, R. Bagnulo, D. C. Loconte, F. C. Susca, N. Resta, C. Simone, The longevity SNP rs2802292 uncovered: HSF1 activates stress-dependent expression of FOXO3 through an intronic enhancer. *Nucleic Acids Res.* **46**, 5587–5600 (2018).

58. M. Soerensen, S. Dato, Q. Tan, M. Thinggaard, R. Kleindorp, M. Beekman, R. Jacobsen, H. E. Suchiman, A. J. de Craen, R. G. Westendorp, S. Schreiber, T. Stevnsner, V. A. Bohr, P. E. Slagboom, A. Nebel, J. W. Vaupel, K. Christensen, M. McGue, L. Christiansen, Human longevity and variation in GH/IGF-1/insulin signaling, DNA damage signaling and repair and pro/antioxidant pathway genes: Cross sectional and longitudinal studies. *Exp. Gerontol.* **47**, 379–387 (2012).

59. Y. Suh, G. Atzmon, M.O. Cho, D. Hwang, B. Liu, D. J. Leahy, N. Barzilai, P. Cohen, Functionally significant insulin-like growth factor I receptor mutations in centenarians. *Proc. Natl. Acad. Sci. U.S.A.* **105**, 3438–3442 (2008).

60. C. Tazearslan, J. Huang, N. Barzilai, Y. Suh, Impaired IGF1R signaling in cells expressing longevity-associated human IGF1R alleles. *Aging Cell* **10**, 551–554 (2011).

61. B. J. Willcox, T. A. Donlon, Q. He, R. Chen, J. S. Grove, K. Yano, K. H. Masaki, D. C. Willcox, B. Rodriguez, J. D. Curb, FOXO3A genotype is strongly associated with human longevity. *Proc. Natl. Acad. Sci. U.S.A.* **105**, 13987–13992 (2008).

62. J. R. Lin, P. Sin-Chan, V. Napolioni, G. G. Torres, J. Mitra, Q. Zhang, M. R. Jabalameli, Z. Wang, N. Nguyen, T. Gao; Regeneron Genetics Center, M. Laudes, S. Görg, A. Franke, A.

Nebel, M. D. Greicius, G. Atzmon, K. Ye, V. Gorbunova, W. C. Ladiges, A. R. Shuldiner, L. J. Niedernhofer, P. D. Robbins, S. Milman, Y. Suh, J. Vijg, N. Barzilai, Z. D. Zhang, Rare genetic coding variants associated with human longevity and protection against age-related diseases. *Nat. Aging* **1**, 783–794 (2021).

63. A. N. Panche, A. D. Diwan, S. R. Chandra, Flavonoids: An overview. *J. Nutr. Sci.* **29**, e47 (2016).

64. K. Pallauf, N. Duckstein, G. Rimbach, A literature review of flavonoids and lifespan in model organisms. *Proc. Nutr. Soc.* **76**, 145–162 (2017).

65. Q. Xu, Q. Fu, Z. Li, H. Liu, Y. Wang, X. Lin, R. He, X. Zhang, Z. Ju, J. Campisi, J. L. Kirkland, Y. Sun, The flavonoid procyanidin C1 has senotherapeutic activity and increases lifespan in mice. *Nat. Metab.* **3**, 1706–1726 (2021).

66. T. H. Wang, W. C. Tseng, Y. L. Leu, C. Y. Chen, W. C. Lee, Y. C. Chi, S. F. Cheng, C. Y. Lai, C. H. Kuo, S. L. Yang, S. H. Yang, J. J. Shen, C. H. Feng, C. C. Wu, T. L. Hwang, C. J. Wang, S. H. Wang, C. C. Chen, The flavonoid corylin exhibits lifespan extension properties in mouse. *Nat. Commun.* **13**, 1238 (2022).

67. V. Brinkmann, N. Ale-Agha, J. Haendeler, N. Ventura, The aryl hydrocarbon receptor (AhR) in the aging process: Another puzzling role for this highly conserved transcription factor. *Front. Physiol.* **10**, 1561 (2020).

68. J. J. McElwee, E. Schuster, E. Blanc, J. H. Thomas, D. Gems, Shared transcriptional signature in *Caenorhabditis elegans* Dauer larvae and long-lived daf-2 mutants implicates detoxification system in longevity assurance. *J. Biol. Chem.* **279**, 44533–44543 (2004).

69. H. Ashida, S. Nishiumi, I. Fukuda, An update on the dietary ligands of the AhR. *Expert Opin. Drug Metab. Toxicol.* **4**, 1429–1447 (2008).

70. M. F. Yueh, Y. H. Huang, A. Hiller, S. Chen, N. Nguyen, R. H. Tukey, Involvement of the xenobiotic response element (XRE) in Ah Receptor-mediated Induction of Human UDP-glucuronosyltransferase 1A1*. *J. Biol. Chem.* **278**, 15001–15006 (2003).

71. Z. Xue, D. Li, W. Yu, Q. Zhang, X. Hou, Y. He, X. Kou, Mechanisms and therapeutic prospects of polyphenols as modulators of the aryl hydrocarbon receptor. *Food Funct.* **8**, 1414–1437 (2017).

72. C. Huang, Y. Chen, T. Zhou, G. Chen, Sulfation of dietary flavonoids by human sulfotransferases. *Xenobiotica* **39**, 312–322 (2009).

73. M. J. Steinbaugh, L. Y. Sun, A. Bartke, R. A. Miller, Activation of genes involved in xenobiotic metabolism is a shared signature of mouse models with extended lifespan. *Am. J. Physiol. Endocrinol. Metab.* **303**, E488–E495 (2012).

74. F. Li, W. Zhu, F. J. Gonzalez, Potential role of CYP1B1 in the development and treatment of metabolic diseases. *Pharmacol. Ther.* **178**, 18–30 (2017).

75. J. W. Mueller, L. C. Gilligan, J. Idkowiak, W. Arlt, P. A. Foster, The regulation of steroid action by sulfation and desulfation. *Endocr. Rev.* **36**, 526–563 (2015).

76. T. J. Visser, Role of sulfation in thyroid hormone metabolism. *Chem. Biol. Interact.* **92**, 293–303 (1994).

77. B. Samuelsson, Arachidonic acid metabolism: Role in inflammation. *Z. Rheumatol.* **50**, 3–6 (1991).

78. B. Wang, L. Wu, J. Chen, L. Dong, C. Chen, Z. Wen, J. Hu, I. Fleming, D. W. Wang, Metabolism pathways of arachidonic acids: Mechanisms and potential therapeutic targets. *Signal. Transduct. Target. Ther.* **6**, 1–30 (2021).

79. S. D. Seidel, G. M. Winters, W. J. Rogers, M. H. Ziccardi, V. Li, B. Keser, M. S. Denison, Activation of the Ah receptor signaling pathway by prostaglandins. *J. Biochem. Mol. Toxicol.* **15**, 187–196 (2001).

80. M. Kniazeva, M. Han, Fat chance for longevity. *Genes Dev.* **27**, 351–354 (2013).

81. E. J. O’Rourke, P. Kuballa, R. Xavier, G. Ruvkun, ω-6 polyunsaturated fatty acids extend life span through the activation of autophagy. *Genes Dev.* **27**, 429–440 (2013).

82. S. Gangemi, L. Pescara, E. D'Urbano, G. Basile, V. Nicita-Mauro, G. Davì, M. Romano, Aging is characterized by a profound reduction in anti-inflammatory lipoxin A4 levels. *Exp. Gerontol.* **40**, 612–614 (2005).

83. N. S. Yarla, A. Bishayee, L. Vadlakonda, R. Chintala, G. R. Duddukuri, P. Reddanna, K. S. V. G. K. Dowluru, Phospholipase A2 isoforms as novel targets for prevention and treatment of inflammatory and oncologic diseases. *Curr. Drug Targets* **17**, 1940–1962 (2016).

84. F.-Y. Zhang, R.-Z. Li, J.-X. Li, X.-X. Fan, C. Xie, L. Liu, X.-J. Yao, E. L.-H. Leung, Phospholipase A_2 as a novel therapeutic target in lung cancer. *Integr. Cancer Sci. Ther.* **7**, 949–959 (2020).

85. J. D. Lambert, N. Yennawar, Y. Gu, R. J. Elias, Inhibition of secreted phospholipase A2 by proanthocyanidins: A comparative enzymological and in silico modeling study. *J. Agric. Food Chem.* **60**, 7417–7420 (2012).

86. G. Man, T. M. Mauro, Y. Zhai, P. L. Kim, C. Cheung, M. Hupe, D. Crumrine, P. M. Elias, M. Q. Man, Topical hesperidin enhances epidermal function in an aged murine model. *J. Invest. Dermatol.* **135**, 1184–1187 (2015).

87. J. S. Kim, Investigation of phenolic, flavonoid, and vitamin contents in different Parts of Korean Ginseng (Panax ginseng C.A. Meyer). *Prev. Nutr. Food Sci.* **21**, 263–270 (2016).

88. W. Hou, J. Pei, Y. Wang, J. Zhang, H. Zheng, R. Cui, Anti-ageing effects of red ginseng on female *Drosophila melanogaster*. *J. Cell. Mol. Med.* **24**, 3751–3755 (2020).

89. M. Baghdadi, D. Karasik, J. Deelen, Genetic control of aging, In: Encyclopedia of gerontology and population aging.

90. D. J. Sauer, B. J. Heidinger, J. D. Kittilson, A. R. Lackmann, M. E. Clark, No evidence of physiological declines with age in an extremely long-lived fish. *Sci. Rep.* **11**, 9065 (2021).

91. R. J. Kinsella, A. Kähäri, S. Haider, J. Zamora, G. Proctor, G. Spudich, J. Almeida-King, D. Staines, P. Derwent, A. Kerhornou, P. Kersey, P. Flicek, Ensembl BioMarts: A hub for data retrieval across taxonomic space. *Database (Oxford)* **2011**, bar030 (2011).

92. J. Herrero, Ensembl comparative genomics resources. *Database* **20**, bav096 (2016).

93. A. Kozomara, S. Griffiths-Jones, miRBase: Integrating microRNA annotation and deep-sequencing data. *Nucleic Acids Res.* **39**, D152–D157 (2011).

94. S. Dimitrieva, P. Bucher, UCNEbase - A database of ultraconserved non-coding elements and genomic regulatory blocks. *Nucleic Acids Res.* **41**, D101–D109 (2013).

95. A. R. Quinlan, I. M. Hall, BEDTools: A flexible suite of utilities for comparing genomic features. *Bioinforma. Oxf Engl.* **26**, 841–842 (2010).

96. J. S. Nelson, T. C. Grande, M. V. H. Wilson, Fishes of the World. Fifth Edit. N. J. Hoboken, John Wiley & Sons, Inc.; (2016).

97. M. Malmstrøm, M. Matschiner, O. K. Tørresen, B. Star, L. G. Snipen, T. F. Hansen, H. T. Baalsrud, A. J. Nederbragt, R. Hanel, W. Salzburger, N. C. Stenseth, K. S. Jakobsen, S. Jentoft, Evolution of the immune system influences speciation rates in teleost fishes. *Nat. Genet.* **48**, 1204–1210 (2016).

98. E. B. Rondeau, A. M. Messmer, D. S. Sanderson, S. G. Jantzen, K. R. von Schalburg, D. R. Minkley, J. S. Leong, G. M. Macdonald, A. E. Davidsen, W. A. Parker, R. S. A. Mazzola, B. Campbell, B. F. Koop, Genomics of sablefish (*Anoplopoma fimbria*): Expressed genes, mitochondrial phylogeny, linkage map and identification of a putative sex gene. *BMC Genomics* **14**, 452 (2013).

99. J. M. Daane, A. Dornburg, P. Smits, D. J. MacGuigan, M. Brent Hawkins, T. J. Near, H. W. Detrich III, M. P. Harris, Historical contingency shapes adaptive radiation in Antarctic fishes. *Nat. Ecol. Evol.* **3**, 1102–1109 (2019).

100. A. M. Bolger, M. Lohse, B. Usadel, Trimmomatic: A flexible trimmer for Illumina sequence data. *Bioinformatics* **30**, 2114–2120 (2014).

101. X. Huang, CAP3: A DNA sequence assembly program. *Genome Res.* **9**, 868–877 (1999).

102. R. C. Edgar, Search and clustering orders of magnitude faster than BLAST. *Bioinformatics* **26**, 2460–2461 (2010).

103. F. J. Sedlazeck, P. Rescheneder, A. Von Haeseler, NextGenMap: Fast and accurate read mapping in highly polymorphic genomes. *Bioinformatics* **29**, 2790–2791 (2013).

104. K. Katoh, D. M. Standley, MAFFT multiple sequence alignment software version 7: Improvements in performance and usability. *Mol. Biol. Evol.* **30**, 772–780 (2013).

105. L. T. Nguyen, H. A. Schmidt, A. Von Haeseler, B. Q. Minh, IQ-TREE: A fast and effective stochastic algorithm for estimating maximum-likelihood phylogenies. *Mol. Biol. Evol.* **32**, 268–274 (2015).

106. K. Chen, D. Durand, M. Farach-Colton, NOTUNG: A program for dating gene duplications and optimizing gene family trees. *J. Comput. Biol.* **7**, 429–447 (2000).

107. H. Li, B. Handsaker, A. Wysoker, T. Fennell, J. Ruan, N. Homer, G. Marth, G. Abecasis, R. Durbin; 1000 Genome Project Data Processing Subgroup, The sequence alignment/map format and SAMtools. *Bioinformatics* **25**, 2078–2079 (2009).

108. J. T. Robinson, H. Thorvaldsdóttir, W. Winckler, M. Guttman, E. S. Lander, G. Getz, J. P. Mesirov, Integrative genomics viewer. *Nat. Biotechnol.* **29**, 24–26 (2011).

109. V. Ranwez, S. Harispe, F. Delsuc, E. J. P. Douzery, MACSE: Multiple alignment of coding SEquences accounting for frameshifts and stop codons. *PLOS ONE* **6**, e22594 (2011).

110. J. Pinheiro, D. Bates, R Core Team, nlme: Linear and Nonlinear Mixed Effects Models. R package version 3.1–157 (2022); https://CRAN.R-project.org/package=nlme

111. E. Paradis, K. Schliep, ape 5.0: An environment for modern phylogenetics and evolutionary analyses in R. *Bioinformatics* **35**, 526–528 (2019).

112. Y. Benjamini, Y. Hochberg, Controlling the false discovery rate: A practical and powerful approach to multiple testing. *J. R. Stat. Soc. Ser. B Methodol.* **57**, 289–300 (1995).

113. M. Ackermann, K. A. Strimmer, A General modular framework for gene set enrichment analysis. *BMC Bioinformatics* **10**, 47 (2009).

114. X. Chen, L. Wang, B. Hu, M. Guo, J. Barnard, X. Zhu, Pathway-based analysis for genome-wide association studies using supervised principal components. *Genet. Epidemiol.* **34**, 716–724 (2010).

115. N. L. Tintle, B. Borchers, M. Brown, A. Bekmetjev, Comparing gene set analysis methods on single-nucleotide polymorphism data from Genetic Analysis Workshop 16. *BMC Proc.* **3**, S96 (2009).

116. C. A. de Leeuw, J. M. Mooij, T. Heskes, D. Posthuma, MAGMA: Generalized gene-set analysis of GWAS data. *PLOS Comput. Biol.* **11**, e1004219 (2015).

117. ADF&G, Mark, Tag and Age Laboratory; https://mtalab.adfg.alaska.gov/ADU/analysis.aspx#/maxage.

118. G. M. Cailliet, A. H. Andrews, E. J. Burton, D. L. Watters, D. E. Kline, L. A. Ferry-Graham, Age determination and validation studies of marine fishes: Do deep-dwellers live longer? *Exp. Gerontol.* **36**, 739–764 (2001).

119. C. P. Archibald, W. Shaw, B. M. Leaman, Growth and mortality estimates of rockfishes (Scorpaenidae) from B. C. Coastal Waters (1981) pp. 1977–1979.

120. J. T. Bennett, G. W. Boehlert, K. K. Turekian, Confirmation of longevity in *Sebastes diploproa* (Pisces: Scorpaenidae) from 210Pb/226Ra measurements in otoliths. *Mar. Biol.* **71**, 209–215 (1982).

121. CARE (Committee of Age-Reading Experts), Manual on generalized age determination procedures for ground-fish (2000); https://care.psmfc.org/about/ageing-manual/.

122. S. C. Meyer, “Composition and biomass of the recreational rockfish *Sebastes* harvest in Southcentral Alaska, 1992–1995.” *Alsk. Dep. Fish Game Fish Data Ser.* (no. 00-6) (2000).

123. AnAge: The animal ageing and longevity database (Human Ageing Genomic Resources, 2017); https://genomics.senescence.info/species/index.html.

124. M. S. Love, J. Butler, *The rockfishes of the northeast Pacific*, M. S. Love, M. Yoklavich, L. Thorsteinson, Eds. (University of California Press, 2002).

125. A. L. Shanks, G. L. Eckert, Population persistence of California current fishes and benthic crustaceans: A marine drift paradox. *Ecol. Monogr.* **75**, 505–524 (2005).

126. R. Wong, “Age and growth of the northern searobin *Prionotus carolinus* (Linnaeus),” thesis, Virginia Institute of Marine Science; http://scholarworks.wm.edu/etd/1539617403.
